# Detection of low-load Epstein-Barr virus in blood samples by enriched recombinase aided amplification assay

**DOI:** 10.1186/s13568-022-01415-9

**Published:** 2022-06-11

**Authors:** Jing-yi Li, Xiao-ping Chen, Yan-qing Tie, Xiu-li Sun, Rui-qing Zhang, An-na He, Ming-zhu Nie, Guo-hao Fan, Feng-yu Li, Feng-yu Tian, Xin-xin Shen, Zhi-shan Feng, Xue-jun Ma

**Affiliations:** 1 Hebei Medical University, No. 361 East Zhongshan Road, Shijiazhuang, 050031 Hebei China; 2 Hebei General Hospital, No. 348 West Heping Road, Shijiazhuang, 050070 Hebei China; 3 NHC Key Laboratory of Medical Virology and Viral Diseases, Chinese Center for Disease Control and Prevention, National Institute for Viral Disease Control and Prevention, No. 155, Changbai Street, Changping District, Beijing, 102206 China; 4 State Key Laboratory for Infectious Disease Prevention and Control, Collaborative Innovation Center for Diagnosis and Treatment of Infectious Disease, Chinese Center for Disease Control and Prevention, National Institute for Communicable Disease Control and Prevention, No. 155, Changbai Street, Changping District, Beijing, 102206 China; 5 North China University of Science and Technology, No. 46 West Xinhua Road, Tangshan, 063009 Hebei China

**Keywords:** M1 beads, Epstein-Barr virus, Sensitive detection, Low viral load, Enrichment, RAA

## Abstract

Epstein-Barr virus (EBV), a common human γ-herpesvirus, infects more than 90% of adults worldwide. The purpose of this study was to establish a novel EBV detection method by combining the recombinase aided amplification (RAA) assay with an initial enrichment step that utilizes magnetic beads coated with a recombinant human mannan-binding lectin (rhMBL, M1 protein). An M1 protein–protein A magnetic bead complex (M1 beads) was prepared and used to achieve separation and enrichment of EBV from blood. After nucleic acid extraction, DNA was amplified by RAA. Using 388 whole blood samples and 1 serum sample, we explored the specificity, sensitivity and applicability of the newly developed detection method and compared it with commercial quantitative real-time polymerase chain reaction (qPCR) following M1 bead enrichment, traditional qPCR and traditional RAA. After enrichment, the positivity rate of EBV was increased from 15.94% to 17.74% by RAA (*P* < 0.05) and from 7.20% to 15.17% by qPCR (*P* < 0.05). The viral loads after enrichment were increased by 1.13 to 23.19-fold (*P* < 0.05). Our data demonstrates that an RAA assay incorporating M1 bead enrichment is a promising tool for detecting low EBV viral loads in blood samples that will facilitate an early response to EBV infection.

**Graphical abstract:**

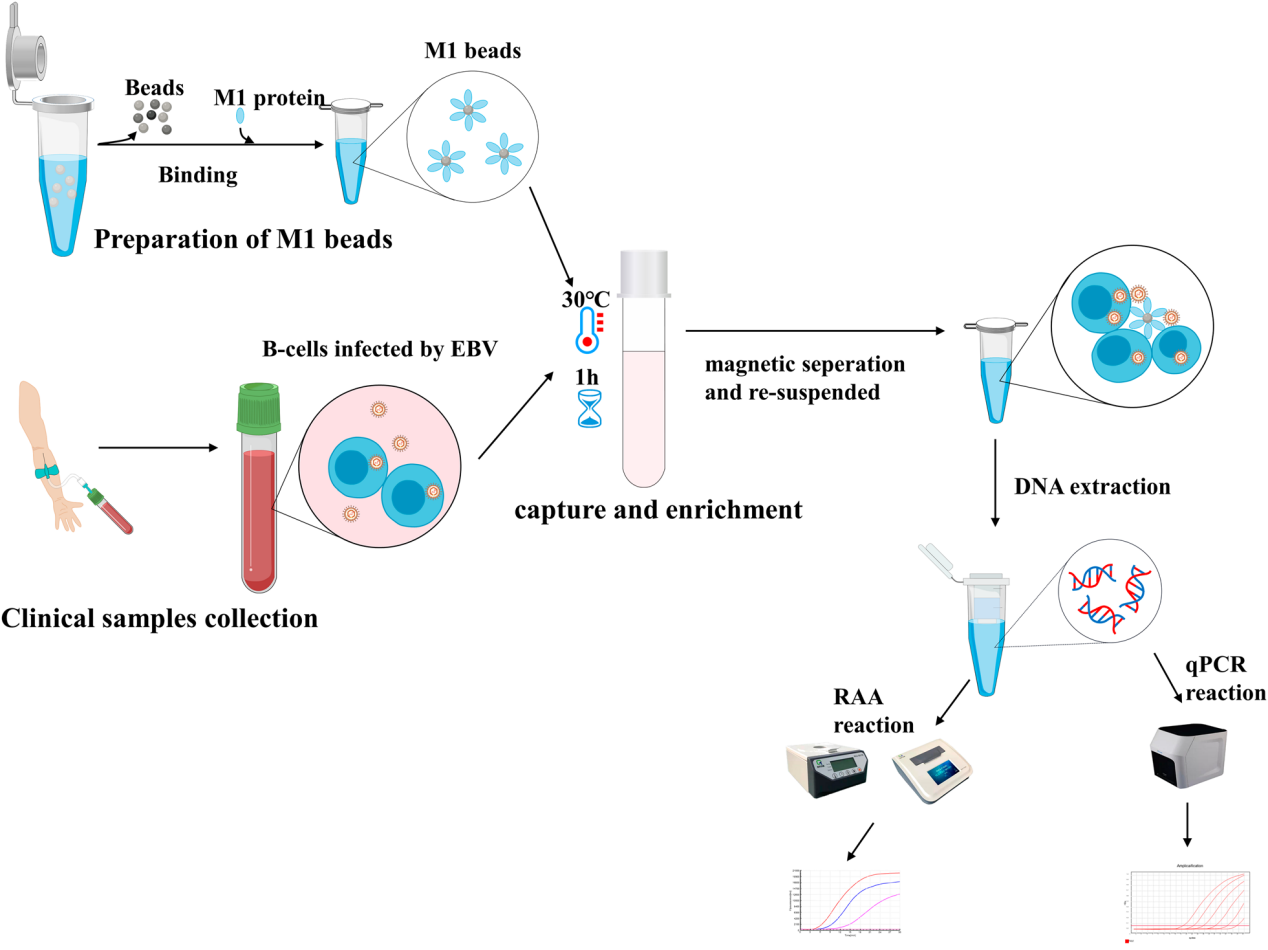

**Supplementary Information:**

The online version contains supplementary material available at 10.1186/s13568-022-01415-9.

## Key points

1. The RAA with an enrichment step that utilizes magnetic beads coated with M1 protein.
2. A very effective method for detecting low-load virus in blood samples.
3. The first report describing virus detection using this method.

**Supplementary Information:**

The online version contains supplementary material available at 10.1186/s13568-022-01415-9.

## Introduction

Epstein-Barr virus (EBV), known as human herpesvirus IV, infects at least 90% of adults worldwide (Nowalk and Green 2016). Primary infection with EBV is associated with acute infectious mononucleosis, whilst persistent infection is associated with chronic disorders, including autoimmune diseases and various types of cancer (Dunmire et al. 2018). Because of the enormous disease burden attributed to EBV infection, an early diagnosis of EBV is crucial for disease control and prevention.

The most common detection methods for EBV are in situ hybridization, serological assays and nucleic acid amplification techniques. Epstein-Barr encoded RNA-in situ hybridization (EBER-ISH) is deemed the gold standard method for the detection of latent EBV infection in tissue sections (Garady et al. 2014). Nevertheless, EBER-ISH requires a biopsy sample and is technically demanding (Weiss and Chen 2013). Serological assays are laborious and time-consuming due to multiple steps, and occasionally generate false-positive results (Niller and Bauer 2017; Tay et al. 2020). Conversely, nucleic acid amplification technology is divided into variable temperature amplification technology and isothermal amplification technology. The classical variable temperature amplification technique is polymerase chain reaction (PCR) (Hübner et al. 2015). Despite its high specificity, sensitivity and efficiency, PCR depends on complicated thermal cycling machines and trained personnel (Ng et al. 2014; Baron et al. 2019). Isothermal amplification technologies include loop-mediated isothermal amplification (LAMP) and recombinase aided amplification (RAA) (Shen et al. 2019; Li et al. 2017). Previously, Seiko Iwata et al. (2006) successfully detected EBV using LAMP. However, this method requires a set of four primers, the design of which is typically complex (Iwata et al. 2006). Our laboratory has established an internal control reference recombinase aided amplification (ICR-RAA) assay for the detection of EBV. The sensitivity of the EBV ICR-RAA assay is 93.33% using extracted DNA and 72.22% using heat-treated DNA. However, the presence of inhibitors in crude blood or serum samples may have significant adverse effects on the ICR-RAA reaction (Gao et al. 2021).

To overcome these drawbacks, we have developed an EBV detection method that combines RAA with an initial EBV separation and enrichment step using magnetic beads coated with a recombinant human mannan-binding lectin (rhMBL, M1 protein) (Chen et al. 2020). To facilitate EBV separation and enrichment, we coupled the protein A-coated magnetic microspheres with M1 protein for non-specific capture of EBV from a large sample volume of blood (1.5 mL). Subsequently, after a nucleic acid extraction step, RAA analysis was performed to monitor the fluorescence signal in real time. Primers and probes designed for the highly conserved regions of EBV were used to rapidly amplify the target sequences. The sensitivity was further improved by increasing the initial sample volume. Because magnetic beads can be separated under a magnetic field, pathogenic microorganisms can be readily separated from complex raw samples. Given the improved sensitivity, this method is of considerable significance in permitting the timely detection of EBV in low-load blood samples.

## Materials and methods

### Primers and probes

All primers and probes sequences have been previously developed and are listed in Table 1 (Gao et al. 2021). The primers and probes were commercially synthesized by Sangon Biotech (Shanghai, China).

**Table 1:** List of primers and probes used in the study

Primers and probes	Sequence 5′–3′		Primer length (bp)
Forward Primer	CTACCTGTGCCGCATGAAACTGGGCGAGACCGA		33
Reverse Primer	CATGTCACAGTAAGGACAGAGAAGTCTGGG		30
Probe	GAACACCTGAGCGTGGTGAAGCCTCTAACGC-[FAMdT]-[THF]-[BHQ1-dT]-CTGTCCACTCCGAA-C3-spacer		48

BHQ, black hole quencher; C3-Spacer, 3′ phosphate blocker; FAM, 6-carboxyforescein; THF, tetrahydrofuran.

### Preparation of M1 protein

The M1 protein was supplied by the Nosocomial Infection Laboratory of the National Institute for Communicable Disease Control Prevention, Chinese Center for Disease Control and Prevention (Chen et al. 2020). The protein concentration was determined and frozen at −80℃. Before use, the frozen protein reagent was thawed at room temperature.

### Preparation of M1 beads

Firstly, 100 μL magnetic beads were added to a 1.5 mL microcentrifuge tube followed by 1 mL phosphate buffered saline (1×) (PBS, 1×). The supernatant was discarded after magnetic separation with a magnetic frame. This step was repeated twice to wash the magnetic beads, then the magnetic beads were re-suspended in 1 mL PBS (1×). To provide a saturating amount of M1 protein required to coat the magnetic beads, 141 μg of M1 protein was added to PBS (1×) to provide a final volume of M1 protein less than one-fifth of the total volume. The specific volume of the M1 protein solution added to the beads depended on the concentration of M1 protein in each batch. Protein A- coated magnetic beads were bound with the N-terminal IgG1 Fc fragment of M1 protein. The samples were suspended on a suspension instrument for 30 min at room temperature to form an M1-protein A magnetic bead complex (M1 beads). After magnetic separation with a magnetic frame, the supernatant was discarded and the M1 beads were washed once with 1 mL of PBS (1×) and then the supernatant was again discarded after magnetic separation with a magnetic frame. Finally, the complexes were re-suspended in 100 μL PBS (1×) solution. The prepared M1 beads were stored at 4℃ for up to 14 days (Chen et al. 2020).

### Capture of EBV virions and preparation of DNA template

For each blood sample, 1.5 mL of whole blood was added to a 14 mL round-bottomed test tube followed by 8.5 mL of PBS (1×). Next, 100 μL of prepared M1 beads were added to the tube with 43.4 μL of 1 mol/L CaCl_2_ and mixed by inversion. The tubes were then put into a 30℃ incubator and suspended in a suspension meter for 1 h. After that, the magnetic beads were adsorbed onto one side of the tube wall by a magnetic rack, and the supernatant was carefully discarded. The M1 beads were subsequently re-suspended in 200 μL of PBS (1×) and stored at −20℃ until further use. Finally, EBV DNA was extracted using the QIAamp DNA Blood Mini Kit (Qiagen, Hilden, Germany) for use as the reaction template for RAA and qPCR.

### RAA reaction

Each tube contained 50 μL of reaction mix. The master mix consisted of 25 μL of rehydration buffer (RAA exo kit), 0.6 μL of 10 μM EBV probe, 2.1 μL of 10 μM forward and reverse primers and 15.7 μL of DNase-free water. The 45.5 μL master mix was added to a lyophilized RAA pellet provided by the RAA exo kit which consisted of the recombinase and single strand DNA-binding protein (SSB). 2.5 μL of 280 mM magnesium acetate was then pipetted into the tube lids. After adding 2 μL of template, the tube was shaken and centrifuged in a constant-temperature centrifuge (QT-RAA-B6100) at 39.0 ℃ for 4 min and then transferred to a real-time fluorescence detection instrument (QT-RAA-F1620; Qitian Biological Co, Ltd) at 39.0 ℃ for 30 min to monitor the fluorescence signal in real time. A negative control (nuclease-free water) was included in each run. Positive values were calculated automatically by the QT-RAA-F1620 instrument after setting the slope K value to be greater than or equal to 20 (Zhang et al. 2019; Wang et al. 2020).

### The detection of clinical samples

A total of 389 samples (1 serum sample and 388 whole blood samples), which were collected from 389 patients at Hebei General Hospital during the period from June 2021 to July 2021. In addition, we collected general data for all cases. The patients were apprised of the study’s purpose and of their right to keep information. All samples were dispensed into 2 mL cryogenic vials and transported to the Central Laboratory of the Chinese Center for Disease Control and Prevention and stored at 4 ℃ until further processing (within 72 h).

To evaluate the applicability of the new developed method, the samples were tested in parallel using RAA following M1 bead enrichment, commercial quantitative real-time polymerase chain reaction (qPCR, DaAn Gene, Guangzhou, China) following M1 bead enrichment, traditional RAA and traditional qPCR. EBV genomic DNA was extracted using the QIAamp Blood DNA Mini Kit (Qiagen, Hilden, Germany), according to the manufacturer’s protocol. To prevent the magnetic beads blocking the spin column, they were separated from the mixture using a magnetic field before the mixture was pipetted to the QIAamp Mini spin column.

### Statistical analysis

The results were analyzed using the paired-sample *t* test, Chi-square test and Kappa test, and a *P*-value less than 0.05 was considered statistically significant. All statistical analyses were performed in SPSS 21.0 (IBM).

## Results

### Application of the RAA assay using M1 bead enrichment to clinical samples and its comparison with qPCR

Of the 389 samples, 69 tested positive for RAA after enrichment. The patients were a combination of patients with respiratory diseases (n = 24), patients with cardiovascular diseases (n = 22), patients with haematological diseases (n = 8), and patients with autoimmune diseases (n = 7). Their disease information is shown in the Additional file 1: Tables S1 and S2. The age of the patients ranged from 1 to 99 years.

The RAA results before and after enrichment are shown in Table 2 and shown that the EBV positivity rate was increased from 15.94% (15.68% + 0.26%) to 17.74% (15.68% + 2.06%) after enrichment. We performed a Chi-square test on the data, and the difference was statistically significant (*P* < 0.05). Using the RAA assay, we also measured the ‘time-to-positivity’ in 61 samples which were positive both before and after enrichment and conducted paired-samples T tests. The detailed data was shown in Additional file 1: Table S3 in the supplementary text. After enrichment, the ‘time-to-positivity’ was shortened and this difference was statistically significant (*P* = 0004, *P* < 0.05).

**Table 2:** Comparison of the RAA assay following M1 bead enrichment with the traditional RAA assay

	The RAA assay following M1 bead enrichment		
Traditional RAA assay		positive (rate%)	negative (rate%)
	positive (rate%)	61 (15.68%)	1 (0.26%)
	negative (rate%)	8 (2.06%)	319 (82.00%)
	*P*	0.039	

**Fig. 1:**
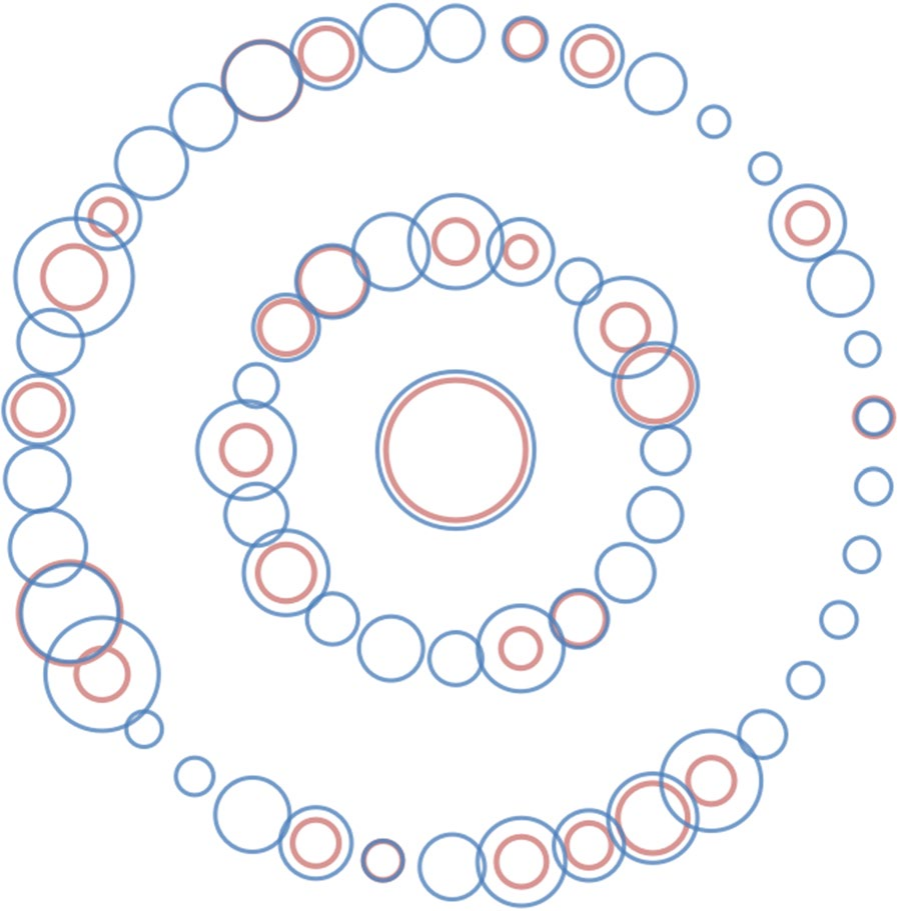
Bubble chart based on the viral load detected by the traditional qPCR and the qPCR assay followed by M1 bead enrichment. The bubble size is the square root of viral load, the number of blue bubbles is the number of positive samples after enrichment, the number of pink bubbles is the number of positive samples before enrichment. The common center of two bubbles shared by the same sample

The results of qPCR before and after enrichment are shown in Table 3. These data revealed that after enrichment, the positivity rate for EBV was increased from 7.20% (7.20% + 0.00%) to 15.17% (7.20% + 7.97%). We also performed Chi-square tests on these data, and the difference was statistically significant (*P* < 0.05). Figure 1 showed the comparison of viral loads before and after enrichment. After enrichment, the viral loads were increased by 1.13 to 23.19-fold and this increase was statistically significant (*P* < 0.05). Among the successfully enriched samples, each sample was enriched in a different fold. The most effective one had a viral load of 236.810 copies/mL before enrichment and a viral load of 5491.920 copies/mL after enrichment, representing an enrichment fold of 23.19. The specimen with the least enrichment effect had a viral load of 74.841 copies/mL before enrichment and 84.377 copies/mL after enrichment, being an enrichment factor of 1.13. The Ct positive cut-off value of the PCR kit was 30, and the detected viral load ranged from 51.8 to 20,158.3 copies/mL (the corresponding Ct value range was 29.6 to 21.2).

**Table 3:** Comparison of the qPCR assay following M1 bead enrichment with the traditional qPCR assay

	The qPCR assay following M1 bead enrichment		
Traditional qPCR assay		positive (rate%)	negative (rate%)
	positive (rate%)	28 (7.20%)	0 (0.00%)
	negative (rate%)	31 (7.97%)	330 (84.83%)
	*P*	< 0.001

## Discussion

In this study, we developed a novel method using a combination of immunomagnetic beads separation and enrichment followed by RAA. Studies have reported that immunomagnetic separation technology is also used for the detection and enrichment of *Listeria monocytogenes, Vibrio parahaemolyticus*, *Mycobacterium tuberculosis* and *Escherichia coli* (Kraft et al. 2017; Intorasoot et al. 2016; Jiang et al. 2020; Stewart et al. 2017; Wadud et al. 2010; Zeng et al. 2014). These researchers used specific antibodies combined with magnetic beads to capture the target pathogen, which was then combined with PCR, LAMP and other techniques to perform nucleic acid amplification. Compared with the traditional culture method, it has been shown that after enrichment with magnetic beads, bacteria can be detected several hours or even several days earlier. In contrast to most studies, we did not use specific antibodies to capture a specific pathogen but instead used M1 protein to non-specifically capture pathogens with an M1 protein binding site from the blood (Chen et al. 2020). In addition, in the subsequent nucleic acid amplification stage, we used a rapid RAA isothermal amplification assay. To our knowledge, this is the first report describing the detection of virus using a combination of M1 bead enrichment and RAA.

Mannan-binding lectin (MBL) is an important anti-infection molecule in human innate immunity (Heitzeneder et al. 2012). The carbohydrate recognition domain (CRD) region of MBL can bind to monosaccharides on the surface of pathogens, thus making it possible to non-specifically capture more than 90 pathogens (Neth et al. 2000; Thiel and Gadjeva 2009). The M1 protein used in this study is a fusion protein constructed by fusing MBL neck region, CRD region and human IgG1 Fc fragment. Due to the retention of the CRD region, the M1 protein also has the same recognition function as MBL. The majority of EBV is hidden in leukocytes but is also present in plasma/serum during the active stage of disease (Shaklawoon et al. 2020). Therefore, the virus can be effectively captured if the leukocytes are enriched. Normally, there is no recognition site for MBL protein on the surface of leukocytes in healthy people (Cooper et al. 2014). Interestingly, our results showed that the ‘time-to-positivity’ of RAA was shortened and the fluorescence value was higher for almost every sample after M1 bead enrichment (Additional file 1: Table S3, Figs. S1 and S2). We speculate that the invasion of EBV might lead to changes in the surface composition of leukocytes, thus exposing an M1 protein recognition site (Garred et al. 2003; Nielsen et al. 1998).

The newly developed method makes full use of the advantages of two commonly used diagnostic tools. Firstly, at the initial immunomagnetic separation (IMS) step, M1 protein can non-specifically capture a variety of pathogens, which is of value when screening for bloodborne infections. Studies have shown that nano-sized magnetic beads have a larger surface area, which can increase the probability of direct contact between ligands and receptors and are therefore suitable for rapid medical diagnostics (Cooper et al. 2014). In addition, magnetic beads are a mobile solid phase carrier, which is conducive to an effective reaction. Magnetic beads have several advantages, including good magnetic responsiveness, high separation efficiency, straightforward operation, no need for large instruments and no risk of damage to the target product (Buchanan et al. 2017). In the current study, the principle of enriching cells using M1 beads allows for leukocytes infected by EBV, as well as cell-free virus in plasma/serum, to be captured by the M1 protein coated magnetic beads. Under the action of an external magnetic field, the magnetic beads can then be isolated. Simultaneously, infected cells and cell-free EBV are also trapped in the magnetic field due to their absorption by M1 protein coated magnetic beads. Other impurities are not captured in the magnetic field because they cannot bind to the M1 protein. By separating that target component from impurities in the sample, their potential for interference in downstream detection can be avoided and the efficiency of subsequent nucleic acid extraction will be improved. This newly developed method makes the test result more accurate and reliable, saves time and material resources and improves work efficiency. In this study, the sample volume was increased to 1.5 mL, increasing the number of pathogens that could be captured; the sensitivity of the method was thus improved. Taking advantage of the rapid speed of the RAA reaction, the combination of sensitivity and rapid detection is realized, and in our study, we were able to obtain results within half an hour following nucleic acid extraction. Compared with other nucleic acid amplification methods, the efficiency is greatly improved.

In this study, we found that the sensitivity was not affected by short-term storage of the beads. Our data demonstrated that the M1 beads could be stored at 4℃ for 14 days. It is recommended that the M1 beads be prepared in advance for later use rather than prepared specifically for each test. Subsequent experiments will aim to optimize these conditions, and in particular, to further improve the stability of the magnetic beads preparation, so that the method can be better applied to on-site detection of pathogens.

It is noteworthy that these samples were collected at the Hebei General Hospital for the purpose of testing blood coagulation parameters and were not originally used to check for EBV infection. However, after screening, we found that 17.7% of patients had an EBV infection. Clearly, greater importance should be placed on EBV infection, which is frequently ignored by clinicians.

The approach to EBV detection described here has several limitations. Firstly, overall, it is more time-consuming (2.5 h) than the traditional RAA method. However, this new method can reduce the risk of false negative results caused by low viral load samples. Therefore, the M1 bead enrichment method is recommended for re-examination of samples suspected of EBV infection but found to be negative by traditional molecular techniques. In addition, this method is only applicable to heparin-anticoagulated whole blood samples and not to Ethylenediamine Tetraacetic Acid (EDTA)-anticoagulated or sodium citrate-anticoagulated samples (Gonzales et al. 2014). Ca^2+^ needs to be added in the enrichment step to promote the binding of M1 protein to pathogens. Unfortunately, Ca^2+^ will bind to EDTA and sodium citrate, thereby undermining the anticoagulant effect and eventually leading to blood coagulation (Salvagno et al. 2013; Christiadi et al. 2018; Scaravilli et al. 2018). EDTA also has the potential to affect the function of magnetic beads. Therefore, EDTA-anticoagulated samples should be avoided. If it is unavoidable to use whole blood with a sodium citrate additive, heparin must be added before the enrichment step to prevent coagulation. In conclusion, the proposed method that combines M1 bead enrichment with an RAA assay is a very effective technique for detecting low-load pathogens in blood samples.

## Supplementary Information

**Additional file 1:** **Table S1.** The EBV-positive patients’ disease information. **Table S2.** The disease information for 330 EBV-negative patients without EBV infection.** Table S3.** Time-to-positivity for 61 samples. **Table S4.** Results of EBV analysis using traditional RAA versus traditional qPCR detection kits.** Table S5.** Results of EBV analysis using the RAA assay versus the qPCR assay following M1 bead enrichment.

## Data Availability

The data and materials are available from the corresponding author on reasonable request.

## Abbreviations

EBV: Epstein-Barr virus
RAA: Recombinase aided amplification
qPCR: Quantitative real-time polymerase chain reaction
PCR: Polymerase chain reaction
rhMBL: Recombinant human mannan-binding lectin
EBER-ISH: Epstein-Barr encoded RNA-in situ hybridization
LAMP: Loop-mediated isothermal amplification
ICR-RAA: Internal control reference recombinase aided amplification
rhMBL: Recombinant human mannan-binding lectin
PBS: Phosphate buffered saline
SSB: Single strand DNA-binding protein
MBL: Mannan-binding lectin
CRD: Carbohydrate recognition domain
IMS: Immunomagnetic separation
EDTA: Ethylenediamine Tetraacetic Acid
