# Supplementary material for: Detection of low-load Epstein-Barr virus in blood samples by enriched recombinase aided amplification assay
Source: AMB Express. 2022 Jun 11;12:71. doi: 10.1186/s13568-022-01415-9 (PMC9188631; doi:10.1186/s13568-022-01415-9)
Supplement: Supplementary file 1 — Additional file 1: Table S1. The EBV-positive patients’ disease information. Table S2. The disease information for 330 EBV-negative patients without EBV infection. Table S3. Time-to-positivity for 61 samples. Table S4. Results of EBV analysis using traditional RAA versus traditional qPCR detection kits. Table S5. Results of EBV analysis using the RAA assay versus the qPCR assay following M1 bead enrichment. [file 13568_2022_1415_MOESM1_ESM.docx]

Additional file text

**Table S1** The EBV-positive patients’ disease information

| Disease type | | Number | Rate |
| --- | --- | --- | --- |
| Respiratory diseases | community-acquired pneumonia(CAP) | 7 | 34.8% |
|  | Pulmonary infection | 8 |  |
|  | Chronic bronchitis | 1 |  |
|  | Chronic obstructive pulmonary disease (COPD) | 3 |  |
|  | Pulmonary occupying lesions | 1 |  |
|  | Poorly differentiated squamous cell lung cancer | 1 |  |
|  | Bronchopneumonia | 1 |  |
|  | Poorly differentiated adenocarcinoma of lung | 1 |  |
|  | Lung abscess | 1 |  |
| Cardiovascular disease | Brain infarction | 12 | 31.9% |
|  | Coronary atherosclerotic heart disease | 10 |  |
| Haematological diseases | Acute promyelocytic leukemia | 2 | 11.6% |
|  | Immune thrombocytopenia(ITP) | 3 |  |
|  | Acute monocytic leukemia | 1 |  |
|  | Iron deficiency anemia (IDA) | 2 |  |
| Autoimmune diseases | Primary Sjogren's syndrome(PSS) | 3 | 10.1% |
|  | Rheumatoid arthritis (RA) | 1 |  |
|  | Systemic lupus erythematosus (SLE) | 2 |  |
|  | Polymyositis(PM) | 1 |  |
| Other types of diseases | Digestive system diseases | 4 | 11.6% |
|  | Nephrotic syndrome | 1 |  |
|  | Amyotrophic lateral sclerosis (ALS) | 1 |  |
|  | Septic shock | 1 |  |
|  | Epistaxis | 1 |  |

**Table S2** The disease information for 330 EBV-negative patients without EBV infection

| Disease type | | Number | Rate |
| --- | --- | --- | --- |
| respiratory diseases | Community-acquired pneumonia(CAP) | 14 | 38.8% |
|  | Pulmonary infection | 39 |  |
|  | Bronchitis | 5 |  |
|  | Chronic obstructive pulmonary disease (COPD) | 16 |  |
|  | Pulmonary occupying lesions | 2 |  |
|  | Poorly differentiated squamous cell lung cancer | 1 |  |
|  | Bronchopneumonia | 1 |  |
|  | Poorly differentiated adenocarcinoma of lung | 1 |  |
|  | Bronchial asthma | 9 |  |
|  | Pulmonary embolism | 5 |  |
|  | Interstitial lung disease | 4 |  |
|  | Acute upper respiratory tract infection | 3 |  |
|  | Pulmonary aspergillosis | 1 |  |
|  | Bronchiectasis | 6 |  |
|  | Respiratory failure | 4 |  |
|  | Encephalorrhagia | 1 |  |
|  | Hypertension | 12 |  |
|  | Thoracoabdominal aortic dissection | 1 |  |
|  | Hyperlipoidemia | 1 |  |
|  | Lung abscess | 1 |  |
|  | Allergic pneumonia | 1 |  |
| Cardiovascular disease | brain infarction | 22 | 16.4% |
|  | Coronary atherosclerotic heart disease | 16 |  |
|  | Encephalorrhagia | 1 |  |
|  | Hypertension | 11 |  |
|  | Dissecting aneurysm of thoracic and abdominal aorta | 1 |  |
|  | Hypertriglyceridemia | 1 |  |
|  | hyperlipoidemia | 2 |  |
| Haematological diseases | Acute promyelocytic leukemia (APL) | 9 | 17.9% |
|  | Acute myelogenous leukemia (AML, M2) | 2 |  |
|  | Immune thrombocytopenia(ITP) | 11 |  |
|  | Hodgkin lymphoma (HL) | 1 |  |
|  | Non-Hodgkinlymphoma (NHL) | 11 |  |
|  | Iron deficiency anemia (IDA) | 3 |  |
|  | Multiple myeloma (MM) | 9 |  |
|  | Myeloproliferative neoplasm | 2 |  |
|  | Chronic myelocytic leukemia(CML) | 1 |  |
|  | Anaplastic large cell lymphoma (ALCL) | 1 |  |
|  | Dysfunction of blood coagulation | 2 |  |
|  | Chronic myelomonocytic leukemia (CMML) | 1 |  |
|  | Acute lymphoblastic leukemia (ALL) | 4 |  |
|  | Anaphylactoid purpura | 2 |  |
| Autoimmune diseases | Primary Sjogren's syndrome(PSS) | 7 | 16.4% |
|  | Rheumatoid arthritis (RA) | 23 |  |
|  | systemic lupus erythematosus (SLE) | 9 |  |
|  | Ankylosing spondylitis (AS) | 2 |  |
|  | Connective tissue diseases (CTD) | 8 |  |
|  | Antiphospholipid syndrome(APS) | 2 |  |
|  | Behçet′s disease (BD) | 1 |  |
|  | Adult onset still disease (AOSD) | 2 |  |
| Other types of diseases | Digestive system diseases | 4 | 10.6% |
|  | Nephrotic syndrome | 4 |  |
|  | Urinary tract infection (UTI) | 1 |  |
|  | Septic shock | 1 |  |
|  | Epistaxis | 5 |  |
|  | Monoclonal gammaglobulinemia (MMG) | 2 |  |
|  | Angina | 1 |  |
|  | Mucocutaneous lymph node syndrome (MCLS) | 2 |  |
|  | Systemic inflammatory response syndrome (SIRS) | 2 |  |
|  | Somatic symptom disorders | 1 |  |
|  | Type 2 diabetes | 2 |  |
|  | Necrtizing fasciitis | 1 |  |
|  | Acute suppurative tonsillitis | 1 |  |
|  | Multiple organ failure | 1 |  |
|  | End stage of malignant tumor | 3 |  |
|  | Pancytopenia | 1 |  |
|  | Leukocytopenia | 3 |  |

**Table S3** Time-to-positivity for 61 samples

| Number | Time-to-positivity before enrichment (min) | Time-to-positivity after enrichment (min) | Number | Time-to-positivity before enrichment (min) | Time-to-positivity after enrichment (min) |
| --- | --- | --- | --- | --- | --- |
| 1 | 10.67 | 0.00 | 32 | 11.67 | 2.00 |
| 2 | 10.33 | 1.00 | 33 | 2.00 | 5.00 |
| 3 | 10.67 | 0.00 | 34 | 12.00 | 2.67 |
| 4 | 10.33 | 1.00 | 35 | 4.33 | 5.00 |
| 5 | 25.00 | 0.00 | 36 | 3.33 | 1.33 |
| 6 | 0.33 | 0.00 | 37 | 0.33 | 4.00 |
| 7 | 5.33 | 0.00 | 38 | 3.33 | 1.00 |
| 8 | 11.33 | 0.67 | 39 | 3.00 | 13.67 |
| 9 | 12.33 | 12.33 | 40 | 1.67 | 0.67 |
| 10 | 12.33 | 5.00 | 41 | 13.67 | 2.67 |
| 11 | 13.00 | 4.00 | 42 | 1.00 | 6.33 |
| 12 | 12.33 | 1.00 | 43 | 3.67 | 0.00 |
| 13 | 2.67 | 0.67 | 44 | 7.33 | 1.00 |
| 14 | 6.00 | 1.67 | 45 | 0.00 | 14.00 |
| 15 | 4.00 | 13.33 | 46 | 0.33 | 0.00 |
| 16 | 2.67 | 0.00 | 47 | 0.00 | 0.33 |
| 17 | 16.67 | 0.67 | 48 | 0.00 | 0.00 |
| 18 | 5.33 | 5.67 | 49 | 0.00 | 0.00 |
| 19 | 11.00 | 12.67 | 50 | 17.33 | 2.67 |
| 20 | 10.67 | 1.33 | 51 | 2.33 | 1.00 |
| 21 | 0.00 | 2.00 | 52 | 5.00 | 0.00 |
| 22 | 25.00 | 0.00 | 53 | 0.33 | 1.00 |
| 23 | 1.33 | 5.33 | 54 | 1.67 | 0.33 |
| 24 | 7.00 | 1.67 | 55 | 1.67 | 0.67 |
| 25 | 0.00 | 0.00 | 56 | 0.00 | 0.00 |
| 26 | 1.33 | 10.33 | 57 | 2.00 | 11.67 |
| 27 | 1.67 | 1.00 | 58 | 3.33 | 4.33 |
| 28 | 5.33 | 5.33 | 59 | 3.33 | 3.00 |
| 29 | 4.67 | 3.33 | 60 | 19.67 | 14.67 |
| 30 | 2.67 | 3.33 | 61 | 0.00 | 0.00 |
| 31 | 13.00 | 6.67 |  |  |  |

The results of RAA and qPCR before and after enrichment were checked for consistency. The results are shown in Table S4 and Table S5. Before enrichment, the RAA and qPCR detection frequencies were generally consistent according to the Kappa coefficient, and the Kappa value was 0.581 (*P*<0.05). After enrichment, the consistency between RAA and qPCR was improved and the Kappa value was 0.907 (*P* <0.05).

**Table S4** Results of EBV analysis using traditional RAA versus traditional qPCR detection kits

|  | Traditional RAA | | |
| --- | --- | --- | --- |
| Traditional qPCR assay |  | positive (rate%) | negative (rate%) |
|  | positive (rate%) | 28 (7.20%) | 0 (0.00%) |
|  | negative (rate%) | 34(8.74%) | 327 (84.06%) |
|  | kappa | 0.581 | |
|  | *P* | <0.001 | |

**Table S5** Results of EBV analysis using the RAA assay versus the qPCR assay following M1 bead enrichment

|  | The RAA assay following M1 bead enrichment | | |
| --- | --- | --- | --- |
| The qPCR assay following M1 bead enrichment |  | Positive (rate%) | negative (rate%) |
|  | positive (rate%) | 59 (15.17%) | 0 (0.00%) |
|  | negative (rate%) | 10(2.57%) | 320 (82.26%) |
|  | kappa | 0.907 | |
|  | *P* | <0.001 | |


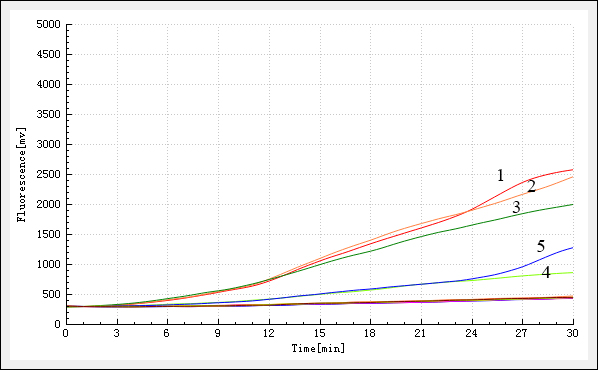


Fig. S1 Amplification curves of RAA assays for Sample 1 to 5 before enrichment


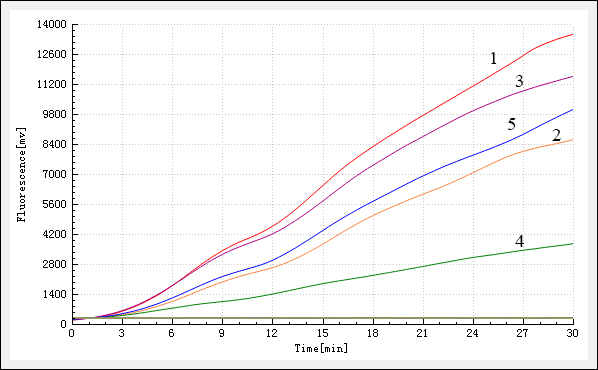


Fig. S2 Amplification curves of RAA assays for Sample 1 to 5 after enrichment
